# Designing a knowledge transfer and exchange strategy for the Alberta Depression Initiative: contributions of qualitative research with key stakeholders

**DOI:** 10.1186/1752-4458-3-11

**Published:** 2009-06-12

**Authors:** Craig Mitton, Carol E Adair, Emily McKenzie, Scott Patten, Brenda Waye-Perry, Neale Smith

**Affiliations:** 1 Faculty of Health and Social Development, University of British Columbia Okanagan, Kelowna BC, Canada; 2 Child and Family Research Institute of British Columbia, Vancouver BC, Canada; 3 Department of Community Health Sciences, University of Calgary, Calgary AB, Canada; 4 Department of Psychiatry, University of Calgary, Calgary AB, Canada; 5 Charis Management Consulting Inc, Edmonton AB, Canada

## Abstract

**Background:**

Depressive disorders are highly prevalent and of significant societal burden. In fall 2004, the 'Alberta Depression Initiative' (ADI) research program was formed with a mission to enhance the mental health of the Alberta population. A key expectation of the ADI is that research findings will be effectively translated to appropriate research users. To help ensure this, one of the initiatives funded through the ADI focused specifically on knowledge transfer and exchange (KTE). The objectives of this project were first to examine the state of the KTE literature, and then based on this review and a set of key informant interviews, design a KTE strategy for the ADI.

**Methods:**

Face to face interviews were conducted with 15 key informants familiar with KTE and/or mental health policy and programs in Alberta. Interviews were transcribed and analyzed using the constant comparison method.

**Results:**

This paper reports on findings from the qualitative interviews. Respondents were familiar with the barriers to and facilitators of KTE as identified in the existing literature. Four key themes related to the nature of effective KTE were identified in the data analysis: personal relationships, cultivating champions, supporting communities of practice, and building receptor capacity. These recommendations informed the design of a contextually appropriate KTE strategy for the ADI. The three-phased strategy involves preliminary research, public workshops, on-going networking and linkage activities and rigorous evaluation against pre-defined and mutually agreed outcome measures.

**Conclusion:**

Interest in KTE on the part of ADI has led to the development of a strategy for engaging decision makers, researchers, and other mental health stakeholders in an on-going network related to depression programs and policy. A similarly engaged process might benefit other policy areas.

## Background

Depressive disorders pose a significant challenge to population health. According to the Global Burden of Disease Project, major depression is the fourth leading contributor to disease burden on a global basis, ranking second in developed countries like Canada [1]. In response to this challenge, the Alberta Depression Initiative (ADI) was launched in the fall of 2004. The mission of the ADI is to enhance the mental health of the Alberta population through research directed against depression. The ADI holds that progress against depression does not depend principally on new genetic or pharmacological discoveries, but rather on ensuring that the benefits of existing knowledge are maximized in the population [2]. In other words, much research evidence on effective care and management already exists and has been extensively confirmed or validated, but has not been fully absorbed into either policy or practice.

Three projects were initially funded by the ADI in 2005/06: 1. a survey of the frequency of depression and associated treatment uptake in Alberta [3]; 2. an investigation of the effects of a depression screening and management protocol for patients with multiple sclerosis in an outpatient clinic population; and 3. a study examining a pharmacist-based intervention to improve patient adherence to antidepressants. In addition, noting a substantial investment of resources by the ADI and challenges observed in translating knowledge into practice, the ADI Project Council felt that translation of results needed to have its own independent research focus. For this reason, a fourth project – reported here – that focused specifically on researching knowledge transfer and exchange (KTE) strategies for the core projects was also funded. KTE refers here to an interactive process involving the exchange of knowledge between research users and researcher producers [4]. The relevance of KTE has grown in recent years as funders demand greater impact for research dollars, researchers seek to have their findings impact decision making directly, and decision makers desire greater defensibility and accountability in making difficult decisions in complex environments.

The objective of the KTE project was to examine the state of the KTE literature and conduct a series of key informant interviews in order to design a KTE strategy for the ADI projects. Findings from the literature synthesis are reported elsewhere [5]. In this paper we outline how the views expressed through the interviews directly informed the design of the KTE strategy. Our informants demonstrate themselves to be highly knowledgeable of KTE as it is described in the existing literature. Their recommendations and the proposed KTE strategy draw upon both this general knowledge and the interviewees' contextually specific understanding of the ADI and the status of mental health policy and programming in Alberta.

In the next section, we describe the research methods. Following this, we describe the stakeholder-recommended features of a KTE strategy for the ADI, as derived from qualitative analysis of the interview transcripts. In the subsequent section, we describe the proposed KTE strategy, noting how it incorporates the respondents' advice. We then reflect upon this strategy and the stakeholders' views in light of the existing literature.

## Methods

The Investigative Team, drawing on its own knowledge and contacts, developed a list of 53 potential informants representing various levels of the health system (e.g. Alberta Health and Wellness, the Alberta Mental Health Board, Regional Health Authorities, community agencies, researchers, research funders and practitioners; consumer groups however were not included at this stage). The intent was to identify a heterogeneous sample of experiential experts from a wide range of backgrounds and interests [6].

All informants were invited to participate by mail. Initial letters were followed by a reminder letter to non-respondents two weeks later. We were able to arrange 15 one-on-one interviews with researchers, decision makers and clinicians whose interests and/or responsibilities were related to KTE generally and/or depression specifically. All but two informants were from Alberta. One-on-one semi-structured interviews were then conducted with each informant, covering respondents' understanding of and experiences with KTE, and specifically KTE in relation to depression in Alberta. Despite a less than desired participation rate (28%), the 15 informants represented a broad cross-section of stakeholders in the health system. They included five policy makers or administrators, three clinicians, three research funders, two academic researchers and two representatives of community agencies or health profession groups.

The interviews were held between September and December 2006, were audio-taped, transcribed verbatim and imported into N*Vivo, a qualitative analysis software package, for coding and analysis. The data were coded inductively with a coding scheme developed through analytic constant comparison [7]. The first step involved labeling 'free nodes', which are basic, not yet categorized themes. Development at the level of free nodes was followed by organization into 'tree nodes', tree structures of category and subcategory. N*Vivo expedited the process of categorization into relationships and themes.

The Behavioral Research Ethics Board at the University of British Columbia and the Conjoint Health Research Ethics Board at the University of Calgary approved the study.

## Results

### Informants' perspectives on KTE

The respondents were asked to speak about their own definition and overall understanding of the concept of KTE, their past experiences (if any) in doing KTE, and to identify barriers to and facilitators of successful KTE (see Appendix 1). Generally, respondents were conversant with key issues in the KTE field and they identified in their own words barriers commonly seen in the literature (see Table 1). Since barriers are already extensively described elsewhere, we do not devote further effort here to analyzing this data. Respondents were also asked to provide details of what a KTE strategy for the ADI might look like. We report their comments in four themes, which arise from our qualitative analysis of the interview transcripts: personal relationships, cultivating champions, supporting communities of practice, and building receptor capacity.

**Table 1:** Barriers to KTE identified in the literature are also cited by ADI key informants

Barriers identified in the literature*	Comments from respondents
Individual level Including:	"I am not sure we have a good system for really gauging relativity of importance and so I am not sure we always put the attention on the things that actually are the most important" (P3)
• Lack of experience and capacity for assessing evidence	"There is no doubt [a] barrier is 'we do everything right and I don't know why you're telling me anyway, because I know. Okay?' [laughter]" (P4)
• Mutual mistrust	"If the people who are going to enact what is recommended, are not ready to do that, then that individual's work is really moot. It made no change" (P5)
• Negative attitude toward change	

Organizational level Including:	"Until, I think, it comes from the top ... and there's an expectation and there's opportunities and there's people hired to do these things specifically, they just don't truly change significantly" (P5)
• Unsupportive culture	"a major problem or a barrier is the protection of interests, and these interests are generally protected by individuals who have got a particular professional orientation or they have got an income stake in the way that things are done" (P1)
• Competing interests	"you don't get rewarded as a research person for any of these kinds of ongoing exchanges or plain language summaries" (P2)
• Researcher incentive system	
• Frequent staff turnover	

Related to communication Including:	"In this day and age, you're inundated with so many different ... getting information isn't a problem. Getting information you need is more the problem" (P9)
• Poor choice of messenger	"[A] big barrier for effective knowledge transfer would be use of appropriate language.... You really have to come up with appropriate language that is customized for the specific audience that you are dealing with" (P1)
• Information overload	"Part of any kind of knowledge transfer is in fact, probably taking a position.... For effective knowledge transfer up, we need to at least say: 'Well, we've got one or two or three options that we're recommending"' (P8)
• Traditional, academic language	"Policy makers want to do something but they would like to have some kind of advice on what do you want me to do, not only present me the finding, the statistics, and then say we need more research to be more sure. You must also provide me with some advice, at least in some direction" (P13)
• No actionable messages (information on what needs to be done and the implications)	

Related to time or timing Including:	"I think the research arena and their processes are also a challenge and their time frames because that whole process is so different from service delivery timelines and processes" (P3)
• Differences in decision makers' and researchers' time frames	"One of the things that really drives the policies is a lot of the times when things become issues for us, things need to be done quickly....I've often had this said to me: 'Surely you want the best evidence.' And my response back is: 'I want whatever you can give me because you do understand that whether you provide the evidence or not, the decision might be made tomorrow'." (P14)
• Limited time to make decisions	

*Source: Reference [5].

#### (A) Effective KTE occurs with the development of personal relationships

Informants argued that effective KTE is built upon long-term, personal relationships between decision makers and researchers. On-going interaction allows both groups to gain better understanding of each others' worlds, and the pattern of incentives and constraints which each face. This can also help ensure that the research questions which are generated and funded are informed by practice needs and can generate results which potentially speak to the matters of greatest concern to the health sector. It is policy maker involvement that enhances the opportunity for effective consideration of policy issues, political limitations, and practical realities.

" [What] is necessary is to have a continual interactive dialogue between the policy maker and the researcher about the question that is being raised and being researched, because as the researcher gets into understanding what some of the background is to the particular topic, that brings up new kinds of information that then informs the policy maker" (P1).

"... you have to have some decision maker, some policy maker, some users of health information attached to your group from day one, so they can (a) help you frame the research questions, (b) tell you how these research questions fit into the policy or decision-making environment and why they are important there, and (c) when you've done your research and when the knowledge is ready to sort of disseminate or transfer, they already have a receptor for that dissemination to happen or for that transfer to happen." (P6)

The close engagement and joint decision making advocated in KTE are also principles of participatory research methods. Researchers who work in this vein may find it easier to achieve the relationships conducive to effective KTE. For instance, one of our respondents, a director of a community agency, described generally disappointing experiences with traditional academic research, but was openly enthusiastic when discussing the trusting relationship they had developed with a participatory action researcher.

"More of the work I have done in the last five years has been involved in participatory research where the researcher knows us, knows the Centre, still has some of the distance to be able to do some of their work, but I think some of the distance in traditional research is artificial, and I think it gets in the way of some of the knowledge transfer. So I think the fact that I have colleagues that I work with and trust, and know me... has made a huge difference." (P15)

#### (B) Organizational leadership and champions are needed to push KTE forward

Some informants suggested that efforts at KTE for the ADI should begin from a thorough mapping of the key players in the mental health arena. "Who are the people in Alberta that are interested in depression – [you] need to come up with an inventory of those" (P1). "You need to know who the actors are here, the organizational actors and the individuals" (P2). "The trick is to make sure you've got a robust inventory of key stakeholders that you can get to very quickly" (P7). Given these comments, respondents presumably believe that no current actor holds such a comprehensive view of the system and its players. Opportunities for linking researchers and decision makers and others, in ways that advance KTE and the use of current research knowledge, might consequently be overlooked. It seems likely to fall to the ADI to undertake such an inventory, though respondents did not suggest exactly how this would be done.

Respondents felt that, from among this group of key players, it would be important to identify the leaders or champions who could help communicate research findings and facilitate KTE. These need not be persons in formal leadership positions.

"When you actually look at what the common denominator is across a whole heterogeneous mix of KTE [pause] a successful KTE initiative, it is very often focused around a single person [pause] who had been charismatic, taken leadership, done championing, so on. So finding those people, identifying champions and leaders for this kind of activity and then being able to resource them, may in fact be the single most effective thing you could do in all of this" (P2).

"You can say what you like in terms of knowledge but generally in terms of practice there are leaders and there are followers and if you get some leaders and champions on side, people's attitudes and behaviors may change" (P12).

#### (C) KTE can be supported through networks and communities of practice

Having a lead person or key contact to manage the interface between researchers and decision makers was felt by some to be a key step: "you need a specific individual identified as your dissemination manager and that individual helps working with the researchers all the way through from the start to the end of the project" (P1).

Respondents noted, however, that it would be insufficient to rely upon single, isolated individuals to advance KTE within decision maker organizations. "I think the grassroots people, they're all over the place, so they don't really have someone to bounce ideas off of in their own Region" (P4). "Putting a single individual into an organization does no good at all; they have to become a magnet and focal point for some new processes and even new structures in the environment if they are going to be effective at doing the knowledge transfer and exchange function" (P2). Thus, they recommended strategies which would foster and grow social networks and communities of practice around new evidence and best practices identified through the ADI.

"Well, first of all you have got to bring together the community of interest..... Number two, you have to identify whether they have a reason to come together and try and advance the practice of health interventions in dealing with depression. Third, bring them together as a community of practice.... Then I think you have got to bring them to the point where they are going to be functioning as a bit of a network..." (P1)

Network, of course, is a somewhat nebulous concept used by the literature in a variety of ways [8,9]. In the sense used by these informants, it refers to people who interact with one another on an on-going basis and who have a common set of interests related to the treatment and management of depression. While this may or perhaps should be formalized in some fashion [8], it does not appear necessary in the respondents' minds to do so. Also in their view, these networks should include not just researchers and health sector organizations like health authorities, but the larger community sector as well. A community agency director, with decades of experience working in a health authority and a university, spoke of the neglect of the community in KTE efforts, despite the knowledge, experience, capacity and interest:

"I think one of the critical pieces here is the exchange strategy including the community, because so often what happens is it only includes the institutions. By that I mean the formal mental health system, the Health Region, and the University... and those of us in the community that are doing the bulk of the work are left out of this. And it is not back and forth. It is usually... 'we, the institution, know and it is you folks in the community that are the recipients'... and sometimes [community agencies] have a lot to offer that the institutions don't." (P15)

#### (D) Organizations need certain capacities in order to take up and use research knowledge

Several respondents noted that successful KTE also depends upon the capacity of decision maker organizations to interpret, contextualize, and use research evidence. This includes dedicated and appropriately skilled personnel:

"We just have not developed the personnel to do this. I mean, it is becoming better but five years ago we really didn't have anybody who specialized in transfer exchange so we are starting to see the emergence of specialists in knowledge transfer and exchange more and more, but it is very, very few of them around. So we have a human resources issue." (P2)

"Having access to the literature is one thing, and having the time to actually do it and then the intellectual capacity to actually sift through it and make some sense of it, that requires some manpower resources" (P10).

It also includes supportive organizational policies and structures: "I think that we do need regional policies in a culture that supports this as a valuable activity" (P3). "I think if you leave it up to individuals to take things on, that's why, I think, most things don't succeed. But if you can get something at an organizational level, for [pause] to be honest, I think, in many cases, things just have to be mandated" (P5).

Several respondents suggested that there were differences between KTE involving research-to-clinical practice, and that involving research-to-policy, with the latter being a more problematic or less understood opportunity. "Less is linked back to policy.... I think [information] gets lost and stays at the clinical or at the scientific level. And they do a good job, moving that information around at that tier. It has a very hard time coming through the glass ceiling though, into the policy world" (P7).

### Proposed KTE strategy

The ADI as a whole adheres to one of the key KTE strategies described here – involving stakeholders throughout the research cycle. The ADI is governed by a multi-disciplinary Project Council that has been actively engaged in the research projects from the outset. As such, policy makers and other stakeholders had involvement in developing the research projects including input on the research questions and study designs.

The aim of the proposed KTE strategy for the ADI is to enable transfer and exchange of information among key stakeholders in order to positively impact depression research, practice, and policy making. In the opinion of our interviewees, effective approaches to KTE need to be carefully thought out and planned in advance

Think the whole chain out – what do you really want to achieve with your message to a policy maker? You must think the whole chain through and not only be clever in putting it, making a summary on one page, or to send it in terms of guidelines, you have to think all the way up to what you want to achieve at the end. And think those steps out and take action on all of them" (P13).

"I think first of all it needs to be something that is clearly developed. It has to have who it is aimed at, who it is targeted at, what are the goals and the objectives" (P12).

This has been confirmed by the literature; see for instance, Lavis et al [10]. We speculate that from the perspective of the ADI, the central goals and objectives would be to increase interaction between key stakeholders, identify relevant research findings that could be taken up in practice and enable an interchange in which decision makers provide feedback on future research activity. Thus we developed the strategy described below, in which we anticipate KTE occurring over three phases.

The centre of the proposed approach is a facilitated workshop of key mental health stakeholders in Alberta, with specific pre- and post-workshop activity. The workshop model was specifically endorsed by several respondents. One interviewee provided a succinct summary of the benefits that such a model would have, in light of the suggested KTE strategies described above:

"I think we are seeing [workshops] more and more and I think that can be very effective. It can be a good use of a fairly small amount of time although if you count up all the hours of the participants it is not insignificant, but you get everyone on the same page pretty quickly, let them know what is happening and then let them work for 4 to 6 hours and you can get a fair bit done. So I think we are tending to want to use that format. I think the other thing it does is put people into face-to-face contact and you can't get the same kind of interaction through other means and I just think the buy in to decisions is much more, the understanding of the complexity of each other's world is much more ... you start to understand the realities of everyone's world [and] then I think that you get some very creative solutions" (P3).

Interactive workshops involving researchers with decision-makers and other stakeholders have been found to be effective in generating new insights and demonstrating the value of close interaction for knowledge translation, for instance in Sabir et al's work on falls prevention in community-dwelling seniors [11]. This is not automatically accomplished, of course, but depends upon good design. Poulos, Zwi and Lord for instance, in a different study of a researcher-policy maker workshop on falls prevention, used observational methods in evaluation which identified strengths and weaknesses in different styles of communication and presentation [12].

#### KTE strategy: phase 1

A one page jargon-free briefing note on each ADI project, set in the context of the broader literature on depression, will be prepared. During a six month period prior to the planned workshop, we will identify the key mental health stakeholders in Alberta, thus as recommended preparing an inventory. The existing degree of interaction among these stakeholders, in relation to the key areas of depression care identified by the ADI, could be assessed at this time through network analysis techniques [13,14]. We will interview a purposively selected subset of these stakeholders, including researchers, policymakers, clinicians, and grassroots community-based organizations. The interviews would: 1. elicit initial response to issues identified in the briefing note and identify specific challenges for change both from policy maker and academic perspectives; 2. determine level of receptivity around behavior changes; 3. identify issues in depression treatment and policy to inform broader discussion about actionable change in phase two; 4. outline existing depression-related research agendas.

#### KTE strategy: phase 2

The second phase will be a two day facilitated workshop led by an external facilitator. The target audience would be the key set of stakeholders identified in phase 1, or representatives of their organizations; those individuals who participated in phase one would be particularly encouraged to attend. The objectives of the workshop will be to 1. foster a high level of interaction between a key set of stakeholders in depression research, policy and practice in Alberta; and 2. understand and grapple with findings from the ADI projects. Workshop participants could jointly develop potential policy changes that may affect multiple levels in the system with identified leads and set timelines. They could also jointly set out an agenda for future depression research, and identify new lines of inquiry for researchers to pursue in partnership with decision makers.

The focus of the workshop itself is on bringing together stakeholders at multiple levels of the system to dialogue, challenge and over time foster change both in academic and health service delivery organizations. In many ways, this specific strategy is meant to be the launching point for longer term networking and relationship building involving both grassroots stakeholders and high-level administrators. On an on-going basis, such a network could be a source of information about depression that could be accessed by the public, health professionals and administrators, and could offer public and continuing professional clinical education around depression practice including screening tools, training sessions and updated practice guidelines. Brief reviews or syntheses of existing research and summaries of the strength of evidence for different interventions may be another product useful to policy makers and practitioners [15]. Efforts and activities to build receptor capacity, or ability to adopt and deploy research evidence effectively, would be another key network role. To enable ongoing evaluation, network structure and membership should be formalized [8]. We are cognizant that a single event is unlikely to change behavior; thus our focus will be as much about fostering dialogue and creating an environment where change is discussed as it is in transferring specific knowledge. This fits with the comments from numerous informants about the need for a community of practice to interact, dialogue and develop creative solutions jointly across levels of the system.

#### KTE strategy: phase 3

After the workshop, the KTE strategy will include follow-up for a period of one year. This would include a bi-monthly newsletter to all stakeholders reporting network developments, potential change action and research program activity. It will also include individual follow-up with a panel of key stakeholders at regular intervals (i.e., 3 times over the 12 month period) and in particular provide opportunities for interaction amongst those individuals with designated responsibility for policy formulation within the health system in order to foster ongoing knowledge support.

Phase 3 also includes evaluation of the KTE strategy; the importance of evaluating the effectiveness of KTE is emphasized by Lavis et al [10]. We propose a pre-post study design with a comparison group (i.e., workshop attendees and network members compared to non-attendees and non-members). We hypothesize, based on the research here and our assessment of the existing literature, that network development will be an appropriate strategy to advance KTE in the ADI. Interviews will be conducted at the end of the 12 month period with identified key stakeholders in both groups to gauge the impact on the KTE strategy on a pre-defined set of measures. Key proposed outcome measures are stages of change [16], level of research utilization [17], degree of organizational change [18], assessed in relation to network connectivity and functioning [14,19,20]. The aim will be to measure change in both practice related to the ADI project findings as well as research agendas in the field of depression.

In addition to interviews, document review can be useful for data collection and evaluation [21]. We will thus examine relevant documents to investigate in greater detail changes to research programs (e.g. research grant submissions) and real changes in the policy and decision making realm. Finally, we would recommend that in the longer term, the network monitor longer term impact including changes in relevant mental health outcomes.

## Discussion

The purpose of the key informant interviews was to understand the perspectives and issues of a particular group of health care stakeholders in a specific context for KTE. We used this as guidance in developing a KTE strategy for the ADI. We feel justified in this approach as the recommendations made by the informants coincide with arguments and suggestions previously found in the KTE literature, as described in our recent systematic review [5].

One important issue identified by our informants, which echoes the literature, is that long term relationship building is a critical factor for success [22,23]. KTE has been judged most effective where it incorporates networking opportunities and relationship building [24]. Interactively engaging key leaders or champions, as recommended by our informants, has also been identified in the literature as another important factor for successful KTE [25,26]. Without some clear expression of support from top-levels of the organization, there will not be the development of any culture supportive of KTE. Mechanisms such as face to face meetings have been used successfully in many instances [27-29].

A key interface role to link these leaders with the research community is the 'knowledge broker' [30-33]. However, such individuals alone are limited in their effectiveness. Our informants' suggestion of building up communities of practice has also been advocated by Norman and Huerta [14], who argue that social network development and the community of practice approach to KTE is suited to situations where knowledge and the practice environment are complex and change rapidly. However, it is important, as we propose, to investigate the network approach more carefully. In Scott and Hofmeyer's assessment, to date in the health systems literature, "applications remain largely metaphorical, tending to skirt the implications of adopting the network approach. ... Problems arise when advocates create "networks" without fully and critically translating knowledge from existing theory into planning and practice innovations" [9]. While seemingly a promising strategy, "the lack of data linking networks to outcomes ... limits understanding of how networks may be mechanisms for addressing complex health issues" (p. 133) [19] – thus the implementation and evaluation strategies proposed for the ADI promise to add to the knowledge base around KTE.

Effective KTE for the mental health arena in Alberta will require the involvement of both policy makers and practice leaders, and there is likely substantial benefit from engaging the wider community and its specific forms of knowledge as well [34]. Participatory action research approaches have proven effective in the past in engaging researchers with decision makers and community members [32] in on-going efforts to bring about evidence-based change in public policy and health service delivery; combining this with network analysis techniques offers significant but largely untested potential [35]. "Networks are complex, members' reasons for being involved in collaborative activity often differ, the context in which they operate is constantly changing and time is needed to develop shared understandings, common goals and trust. For these reasons action research seems to be a particularly useful tool for the research into and development of networks" (p. 7) [36].

Finally, we note that many studies have pointed to the need to address not only individual but system-level capacity, through organizational systems and structures [27,34]. It is probable that the most effective KTE requires higher level organizational change, along with structures and institutionalized processes [17]. Our proposed KTE strategy is in this regard limited in its scope or influence of control. Implementing organizational changes – to culture, structure, or process – in mental health policy and service organizations is not realistically actionable by the ADI KTE strategy. For example, the degree of personnel changes within a given organization and the political climate are important factors in knowledge uptake but clearly cannot be substantially influenced by an individual research team.

## Conclusion

In our view the proposed KTE strategy fits with the available evidence on KTE practices and builds on both the qualitative survey findings and Investigative Team experience in the mental health field in Alberta. Our main hypothesis is that KTE will be served through the deliberate cultivation of a province-wide (and beyond) network, including researchers, senior policy makers, clinicians, grassroots advocates and others; such networking builds relationships, fosters champions, and can build the receptor capacities needed for policy and service organizations to take up established and relevant research evidence into practice. A decision to implement the proposed strategy, and allocating resources to fund it, rests with the ADI Project Council. Other groups may want to take a similar approach in designing KTE strategies through reviewing the relevant literature, consulting with local stakeholder groups and including a mechanism to rigorously evaluate the activity against a pre-defined set of outcomes. Over time, in building up the evidence base, future KTE endeavors will be able to be informed by past practice, thereby fostering greater likelihood of a positive return on limited health research funding.

## Competing interests

The authors declare that they have no competing interests.

## Appendix 1

Interview schedule

1. Could you please describe your role in relation to depression in Alberta?

2. Please describe your understanding of knoweldge transfer and exchange.

3. Have you participated in knowledge transfer and exchange activities in the past? If yes, please desribe your experiences.

4. Please describe some challenges to effective knowledge transfer and exchange between decision makers and researchers.

5. Please describe your experience with facilitators to successful knowledge transfer from researchers to decision makers.

6. Please describe your experience with facilitators to successful knowledge transfer from decision makers to researchers.

7. Could you please describe your experiences with a specific approach to knowledge transfer and exchange (i.e. website, knowledge broker, researcher/decision-maker workshop). The benefits? The disadvantages?

8. Noting that we are developing a knowledge transfer strategy to assess how knowledge generated about depression in Alberta (through the ADI) could be integrated with relevant decision making processes, is there anything else that you could tell us to help us in this process?

## Authors' contributions

The initial research conception and design were developed by CM, CA, SP and BWP. Background research and literature review and synthesis were conducted by EM, with assistance of CM, CA, SP and BWP. Participant selection and recruitment was carried out by CM, CA, SP and BWP. EM collected the data and conducted initial analyses. Further data analysis and interpretation was carried out by NS. EM, NS, and CM wrote the initial draft of the text. All authors reviewed and approved the final draft of the text.
